# Effects of Drying Treatments on Nutritional Compositions, Volatile Flavor Compounds, and Bioactive Substances of Broad Beans

**DOI:** 10.3390/foods12112160

**Published:** 2023-05-26

**Authors:** Si Li, Fangwei Liu, Mulan Wu, Yuhao Li, Xiaoxiao Song, Junyi Yin

**Affiliations:** State Key Laboratory of Food Science and Resources, China-Canada Joint Laboratory of Food Science and Technology (Nanchang), Key Laboratory of Bioactive Polysaccharides of Jiangxi Province, Nanchang University, Nanchang 330047, China

**Keywords:** broad beans, drying method, nutrition composition, bioactive substances

## Abstract

In this study, different drying methods, including hot air drying, sun drying, and freeze drying were employed to dry fresh broad beans. The nutritional composition, volatile organic components and bioactive substances of the dried broad beans were systematically compared. The results indicated significant differences (*p* < 0.05) in nutritional composition, such as protein and soluble sugar content. Among the 66 identified volatile organic compounds, freeze drying and hot air drying significantly promote the production of alcohols and aldehydes, while sun drying effectively preserves esters. In terms of bioactive substances, broad beans dried by freeze drying exhibit the highest total phenol content as well as the strongest antioxidant capacity and gallic acid, followed by sun drying. The chemometric analysis revealed that the bioactive compounds in broad beans dried by three different methods were primarily composed of flavonoids, organic acids, and amino acids with significant differentiation. Notably, freeze-dried and sun-dried broad beans exhibited a higher concentration of differential substances.

## 1. Introduction

Broad bean (*Vicia faba* Linn), an annual herbaceous plant belonging to the Leguminosae family, is a highly important crop widely cultivated and consumed in Middle Eastern countries due to its abundant nutrients, such as protein, carbohydrates, and vitamins [1]. Moreover, broad beans are richer in phenolic compounds than other legumes and possess high antioxidant capacity (AC) that can help reduce the risk of cardiovascular disease [2]. However, the presence of free water molecules poses a challenge for their storage and preservation.

Drying is one of the most popular pre-treatment methods for food preservation [3]. In previous studies on beans aimed at investigating the effects of hot air drying (HAD), infrared drying (IRD), microwave drying (MWD), and freeze drying (FD) on nutritional composition attributes [4,5]. Various technologies have their own advantages and disadvantages. Among them, HAD is the most common dehydration process [6], but it has limitations such as nutrient loss and high energy consumption because of long processing time [7]; FD is a newly emerged drying method for maximizing the retention of color, fragrance, and nutrients, but its disadvantage of high consumption has limited its applications in agriculture and business [8]. Furthermore, sun drying (SD) is widely applied because of its low-cost and convenient operation, while it is time-consuming and relies on weather conditions. However, the effects of the various drying methods on nutrients and bioactive substances are different [9]. Moreover, most of the conducted studies limited knowledge of how different drying methods affect the bioactivity of products to a few phytochemical substances. In terms of color parameters, the closest drying method to fresh thyme leaves was natural drying, which effectively preserved chlorophyll concentrations [10]. Liu et al. found that the transformation of bound phenolics was caused by increasing drying temperature [11]. Yang et al. found that HAD promoted the lignification of L. edodes [12]. Politowicz et al. found that the best dehydration methods in the highest total concentrations of volatile compounds with intensity of key sensory attributes were freeze drying and convective drying at −80 °C for shiitake mushrooms [13]. Moreover, in addition to non-volatile components, many studies have investigated the effect of drying methods on volatile components (VOCs). The drying process will also change the proportion of volatile organic compounds with fruit and green aroma, such as (E)-2-hexenal, (Z)-2-heptanal, benzaldehyde, and (E)-2-nonenal [14]. The pepper content of pepper and the antioxidant properties of the beans were all better after FD [4,15]. More comprehensive studies were performed by Pachura et al., who investigated the effect of drying methods, including convective drying, vacuum-microwave drying, and combined drying consisting of convective pre-drying and vacuum-microwave finish-drying on bioactive volatile constituents of sage [16]. To the best of our knowledge, there is a paucity of research on the impact of drying methods on broad beans’ drying characteristics, including bioactive compounds and volatile organic compounds (VOCs). [17]. Hence, the optimal selection and regulation of drying techniques based on nutrient content in conjunction with a more comprehensive profile of bioactive compounds and VOCs pose a significant challenge.

The latest research on untargeted analysis has demonstrated the potential for generating a comprehensive chemical profile through the use of advanced analytical tools, such as ultraperformance liquid chromatography–quadrupole time-of-flight mass spectrometry (UPLC–Q-TOF/MS), gas chromatography-time-of-flight mass spectrometry (GC-TOF/MS), and nuclear magnetic resonance (NMR) [18,19,20,21]. Chemometrics tools have been widely used in food characterization [22]. PCA, Cluster Analysis (CA), and Linear Discriminant Analysis (LDA) have been used to classify green teas according to their geographical origin based on their trace elements [23]. According to previous research, the majority of chemometric methods differentiate broad bean products based on factors such as variety, heat treatment, and harvest time. Currently, the impact of nutrients and small molecules (such as chlorophyll levels, and antioxidant properties) on broad beans has been primarily studied in relation to cultivation conditions and certain geographical environments [24,25,26]. However, there have been limited investigations into the comparison of various drying methods for fresh broad beans in conjunction with chemometric techniques. Additionally, it remains unclear to what extent these different approaches may impact the nutrient composition of broad beans, and whether chemical metrology tools can serve as a reliable basis for discernment and clarification.

This study investigated the impact of three drying methods on the nutritional composition, volatile organic compounds (VOCs), total phenol content (TPC), and antioxidant capacity (AC) of broad beans. Additionally, bioactive substance analysis using UPLC-Q-TOF-MS was conducted, including principal component analysis (PCA) and partial least squares-discriminant analysis (PLS-DA), to further investigate the separation effect and substances responsible for differences in the drying methods on broad beans. The aim of this study is to provide valuable information on large-scale industrial applications of drying techniques.

## 2. Materials and Methods

### 2.1. Materials

Fresh broad beans (*Vicia faba* Linn) were obtained in the Dingxi county of the Gansu province in June 2020. They were stored at 4 °C until used in the further experiments. The whole broad beans, with the pod removed manually were treated by varied drying methods.

### 2.2. Chemical Agents

Folinol, gallic acid, and rutin were purchased from Shanghai Yuanye Biological Co., LTD (Shanghai, China). Phenol was purchased from Aladdin Biotechnology Inc (Shanghai, China). Formic acid was obtained from Thermo Fisher Technologies Co., Ltd. (Waltham, MA, USA), and methanol was purchased from Merck Chromatographic Reagent Co., LTD (Darmstadt, Germany). Hydrochloric acid, sulfuric acid, petroleum ether (30~60 °C), copper sulfate, potassium sulfate, boric acid, sodium nitrite, sodium carbonate, aluminum chloride, sodium hydroxide, and other chemical reagents were analytical-pure and purchased from Xilong Scientific Co., Ltd. 1, 1-diphenyl-2-trinitrophenylhydrazine (DPPH) was purchased from Sigma Company in the United States. RRAP and ABTS kits were obtained from Biyuntian Biotechnology Co., Ltd. (Shanghai, China). The total starch assay kit was obtained from Megazyme International Ireland, Bray Business Park, Bray, Co. Wicklow, Ireland.

### 2.3. Drying Process

Hot air drying (HAD): HAD was performed in the convective oven (model GZX-9420MBE, Shanghai Boxun Experimental Co., Ltd., China) at a temperature of 40 °C for 41 h where the fresh broad beans were uniformly distributed over stainless steel trays. The measurement of the moisture content of the dried broad beans was completed when the beans’ moisture content reached 10 ± 1%. The experiments were conducted at 40 °C to maintain nutritional quality. The end point was 10.49 ± 0.11 (%) of the moisture content.

Sun drying (SD): SD was performed in a ventilated open area with the temperature at 30~35 °C for 34 h; the fresh broad beans were spread uniformly in a direct sunlight environment. The measurement of the moisture content of dried broad beans was completed when the beans’ moisture content reached 10 ± 1%. The end point was 9.80 ± 0.12 (%) of the moisture content.

Freeze drying (FD): FD took place at a freeze-dryer (model RS-232, Labconco Corporation, America) at −80 °C and 1.370 mbar for 68 h (the duration of an automatic run) after overnight pre-freezing in a −80 °C refrigerator. The end point was 5.68 ± 0.18 (%) of the moisture content.

After that, all dried broad beans were broken up using a blade mixer and conducted with 60 mesh screens.

### 2.4. Scanning Electron Microscopy (SEM) Experiments

The microstructure of dried broad beans was determined by using a scanning electron microscope at the center plane of the piece. The SEM observation was taken at 500× and 7000× magnification with an accelerating voltage of 15 kV.

### 2.5. Determination of Nutrition Composition

An automatic multi-sample moisture content analyzer (model prepASH229, Precisa Corporation, Switzerland) was used to determine the moisture content. Samples (0.5~1 g) were taken into the separator at 105 °C for 120 min, and were weighed every 10 min until constant weight.

The ash content was determined by the AOAC 930.05 method. Total starch analyses were performed according to AOAC 996.11 [27]. Protein content was determined by AOAC 920.87 with a nitrogen-to-protein conversion factor of 6.25. The phenol-sulfuric acid method was used for determination of the soluble sugar content [28].

The determination of the nutrition composition could be calculated on the basis of the deduction of moisture content.

### 2.6. Analysis of Volatile Components by Headspace Solid-Phase Microextraction–Gas Chromatography–Mass Spectrometry (HS-SPME-GC–MS)

#### 2.6.1. HS-SPME Extraction of Volatile Components

2 g of dried broad beans powder was weighed and placed in a headspace glass vial, and equilibrated in a water bath at 80 °C for 30 min. SPME fibers (50/30 μm) were inserted into the headspace of the sample and extracted for 30 min.

#### 2.6.2. Identification of Volatile Components by GC–MS

VOCs were qualitatively determined using a GC–MS system (model 7890-7000A, Agilent Corporation, Santa Clara, CA, USA) equipped with a HP-5MS column (30 m × 0.25 mm, 0.25 µm). The injection temperature was maintained at 250 °C, and the carrier gas was He with a flow rate of 1.0 mL/min. The initial column temperature was 35 °C for 0 min; this was ramped up to 75 °C at 2 °C/min for 0 min; then the column temperature was ramped up to 83 °C at 0.5 °C/min for 0 min; then it was ramped up to 90 °C at 7 °C/min for 0 min, followed by an increase to 230 °C at 5 °C/min for 0 min. The total analysis time was 56 min. A triple quadrupole mass spectrometer operated in full scan mode with an ion source at 230 °C and an ionization voltage of 70 eV. The mass collection range was 30–550 *m*/*z*. The volatile compounds were identified by mass spectra from the NIST 17.0 database. The quantification of the identified sage aroma profile constituents was carried out.

### 2.7. Determination of Bioactive Substances

#### 2.7.1. Extraction

The extraction of broad beans used for the measurement of TPC and AC were obtained according to the protocol of Somporn et al. [29]. 1 g of dried broad beans powder was mixed with 8 mL methanol/water (80:20 *v*/*v*) for 30 min, and an ultrasonic water bath was used for improving the extraction efficiency. After centrifugation of the mixed system at 4500 rpm for 10 min, the supernatant was collected and mixed with the solution of washing residue, which was performed twice. The broad beans were extracted and kept at −4 °C until being analyzed for the following indicators.

#### 2.7.2. Total Phenol Content (TPC)

The determination of total phenol content was based on a modified Folin-Ciocalteu method of Cheong et al. [30]. Gallic acid was used as a standard sample, ranging from 5 to 100 mg/L. Of this extract, 200 µL was mixed with 200 µL of Folin-Ciocalteu’s reagent in a centrifuge tube, and kept at least 30 s in room temperature; 600 µL of 10% (*w*/*v*) sodium carbonate was added into the reaction mixture and reacted for 30 min in room temperature. The TPC was determined at the absorbance of 764 nm. The result was expressed in mg GAE/g DW.

#### 2.7.3. Determination of ABTS Free Radical Scavenging Capacity

The determination of ABTS was performed as described in the kit instructions. An aliquot of 170 µL of ABTS reagent was mixed with 10 µL of above extract. The mixture was well mixed and placed in the dark for 6 min. The absorbance at 405 nm was measured. A standard curve was prepared from 100 mM to 800 mM of Trolox. The result was expressed in µmol Trolox/g DW.

#### 2.7.4. Determination of FRAP Assay

The determination of the FRAP assay was based on a modified method of Yu et al. [31]. The FRAP reagent was prepared by mixing 10 mM of TPTZ solution, 20 mM ferric chloride, and 0.3 mM acetate buffer according to 10:1:1. The solution was then incubated at 37 °C for 5 min. An aliquot of 185 µL of FRAP reagent was mixed with 15 µL of above extract. The mixture was well mixed and placed in the dark at 37 °C for 5 min. The absorbance at 593 nm was measured. A standard curve was prepared from 0.15 mM to 1.5 mM of FeSO_4_. The result was expressed in µmol FeSO_4_/g DW.

#### 2.7.5. Determination of DPPH Free Radical Scavenging Capacity

The determination of DPPH free radical scavenging activity was based on a modified method of Cheong et al. [30]. An aliquot of 175 µL of DPPH solution (150 µmol/L) was mixed with 25 µL of above extract. The mixture was well mixed and placed in the dark at room temperature for 3 h. The absorbance at 517 nm was measured. A calibration curve against concentration of standard Trolox in the concentration range 50–500 µmol/L was prepared. The result was expressed in µmol Trolox/g DW.

#### 2.7.6. Analysis of Bioactive Substances by UPLC-Q-TOF-MS

The identification of bioactive substances using UPLC-Q-TOF-MS analysis (model Agilent 6538, Agilent Corporation, Santa Clara, CA, USA) was carried out by an Agilent 1290 HPLC system equipped with a Zorbax Eclipse Plus-C18 (100 × 2.1 mm, 1.8 µm, Agilent, Santa Clara, CA, USA) column at 35 °C. The mobile phase consisted of 0.1% formic acid in water (A) and 0.1% formic acid in methyl alcohol (B). The gradient elution was run as follows: keep 10% B over 2 min, from 10% to 25% B over 6 min, from 25% to 40% B over 4 min, from 40% to 75% B over 4 min, from 75% to 89% B over 23 min, from 89% to 95% B over 2 min, from 95% to 10% B over 3 min, then keep 10% B over 3 min. The sample injection volume was 5 µL. The flow rate was delivered at 0.3 mL/min under a gradient program.

The class separation of components was performed by a Q/TOF mass spectrometer with an ESI interface. The negative ion mode was adopted for samples, and MS data were observed in the range of 50–1700 *m*/*z*. The parameters of mass spectrometry were as follows: capillary voltage, 4.0 kV; drying gas (N_2_) flow rate was 10.0 L/min at 350 °C; nebulizer, 40 psig; pyrolysis voltage, 175 V; collision energy of parent ion, 15 V, injection cone voltage 35.0 V, de-solvation flow (N_2_) velocity 900 L/h, ion source temperature 150 °C.

UPLC-Q-TOF-MS data were perceived depending on the Agilent MassHunter Qualitative Analysis 118 (version B. 05. 00) with peak detection. The data analysis of chromatographic information after processing used Metaboanalyst 5.0, which is a web-based metabolomics data processing tool. Metaboanalyst 5.0 processed the data. The minimum value was used to replace all missing values in order to remove features where the missing value exceeded 50%. Method stability was evaluated using a characteristic ion peak of > 25% RSD within QC. Data normalization took place through normalization by sum, log transformation, and auto scaling to perform (PCA). The normalized data after processing by Metaboanalyst 5.0 was imported into SIMCA 14.1, and then a multivariate analysis of PLS-DA was carried out.

#### 2.7.7. Determination of Phenolic Compounds by UPLC-QQQ-MS

The detection of phenolic compounds using UPLC-QQQ-MS analysis (model Agilent 1290–6460, Agilent Corporation, Santa Clara, CA, USA) was carried out by an Agilent 1290 HPLC system equipped with a Zorbax Eclipse Plus-C18 (100 × 4.6 mm, 1.8 µm, Agilent, Santa Clara, CA, USA) column at 35 °C. The mobile phase consisted of acetonitrile (A) and 0.1% formic acid in water (B). The gradient elution was run as follows: from 10% to 45% A over 8 min, from 45% to 80% A over 8 min, from 80% to 100% A over 4 min, keep 100% A over 3 min, from 100% to 0 A over 3 min. The sample injection volume was 5 µL. The flow rate was delivered at 0.3 mL/min under a gradient program.

The chromatograph was coupled to a triple quadrupole mass spectrometer with an ESI interface. The ESI source worked in negative ion and multiple reaction monitoring modes.

### 2.8. Statistical Analysis

All results were displayed as the mean ± one standard deviation of three replicates. Statistical analyses were presented as mean values standard error estimation using SPSS Statistics software version 17.0 (SPSS Inc., Chicago, IL, 300 USA). The significant differences in the sample were analyzed at *p* < 0.05 level.

## 3. Results

### 3.1. Imaging of Microstructure Using the Scanning Electron Microscope

**Figure 1:**
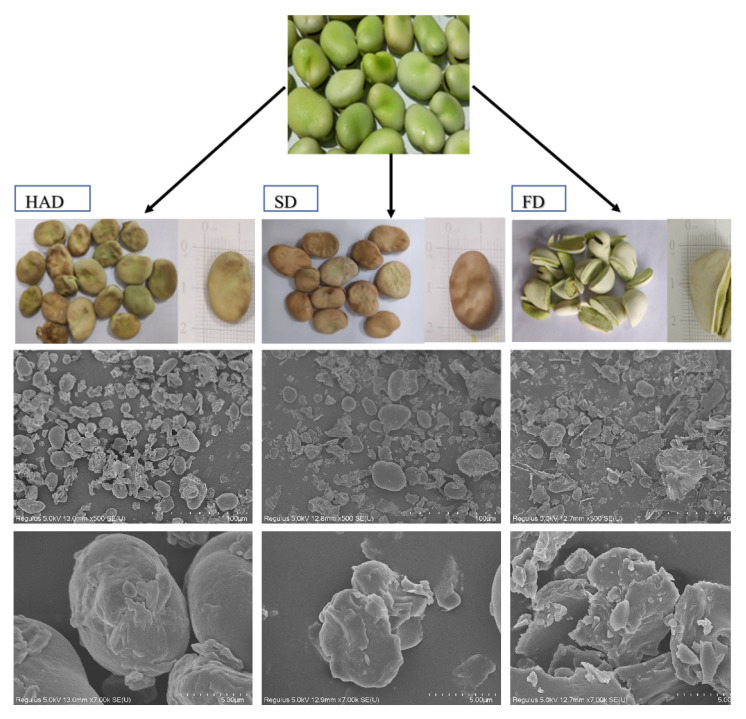
Fresh, dried sample and micro-morphology of HAD, SD, FD dried broad beans. (HAD: hot air drying; SD: sun drying; FD: freeze drying).

Figure 1 exhibits the fresh, dried sample and micro-morphology of the broad beans. The larger particles with uneven and irregular size distribution were in the broad beans treated by FD, while smaller and regular particles were in the broad beans treated by SD and HAD. The smaller particles indicated that the broad beans of SD and HAD more easily powdered. The collapsed cell structures and compact tissues were revealed in the broad beans treated by SD and FD, and an expansive particle structure was revealed in the broad beans treated by HAD, which may have been caused by starch expansion due to weak uniformity of higher temperature [32].

### 3.2. Nutrition Composition Analysis

Table 1 shows the effect of various drying methods on the nutrition composition of dried broad beans, including the content of moisture, ash, starch, resistant starch, protein, and soluble sugar. There were no significant differences of ash, starch or resistant starch content among the different samples (*p* > 0.05); FD could be used to preserve starch (40.43 ± 0.38 g/100 g). Yet, the protein content of broad beans was affected by drying methods to varying degrees. The HAD group showed the highest protein content of 35.07 ± 0.12 g/100 g among all the samples, while the SD and FD group exhibited the lowest amount of protein, possibly due to prolonged exposure to air; the protein content of the FD group was also affected by prolonged pre-freezing in a −80 °C refrigerator. However, Raza et al. [33] reported finding that the protein content of dates had no significant difference in oven-drying and spray-drying. In addition, the SD group showed the highest soluble sugar content (18.30 ± 0.02 g/100 g sample), while the FD group (15.30 ± 0.28 g/100 g sample) had the lowest. This is in accordance with Chen et al.’s work, polysaccharides dried by FD appeared to decrease in glucose content compared with HAD and SD [34].

Evaluating the different methods of drying by merely comparing the nutrient ingredients was not accurate, and other substances that affect the quality of broad beans may also change.

### 3.3. Amino Acids Analysis

The amino acid composition and content of the broad beans after three different drying treatments are shown in Table 2. Except for Try, which was destroyed by acid hydrolysis and could not be detected, 17 amino acids were detected in the dried beans, including 7 essential amino acids and 10 non-essential amino acids, and the total amino acid contents of the beans with different drying treatments ranged from 296.07 to 302.35 mg/g. The amino acid composition content showed that the broad beans had the highest glutamic acid content, followed by Asp, Leu, and Lys. In addition, the results found that FD has a better preservation effect on total amino acids (302.35 mg/g). The lowest total amino acid content of the HAD-treated broad beans may be due to the occurrence of the Maillard reaction. However, the above-mentioned HAD showed an increase in soluble sugar content, and it is possible that the glucose decomposed from the sucrose breakdown masked the Maillard reaction [35]. While the amino acid content of most SD was higher than that of HAD, Asp was more specific, and some studies have shown that Asp is considered to be the least active amino acid in the Maillard reaction [36].

### 3.4. Identification of Volatile Components (VOCs)

**Figure 2:**
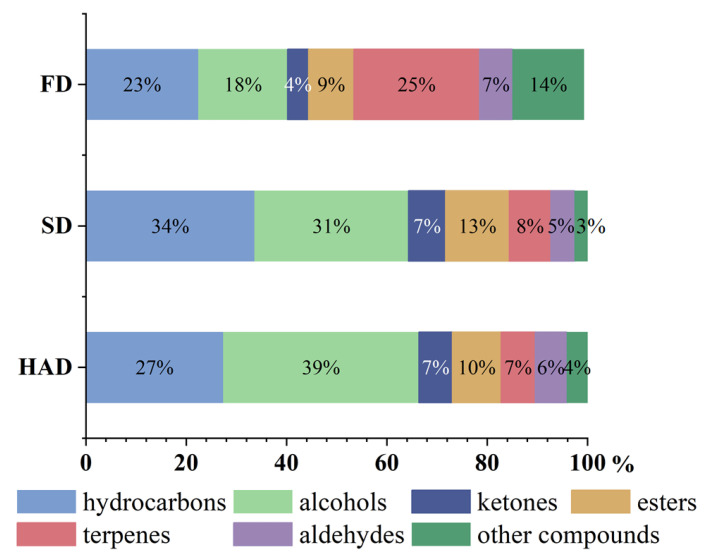
Relative percentages of volatile component classes for three different drying methods of broad beans.

**Figure 3:**
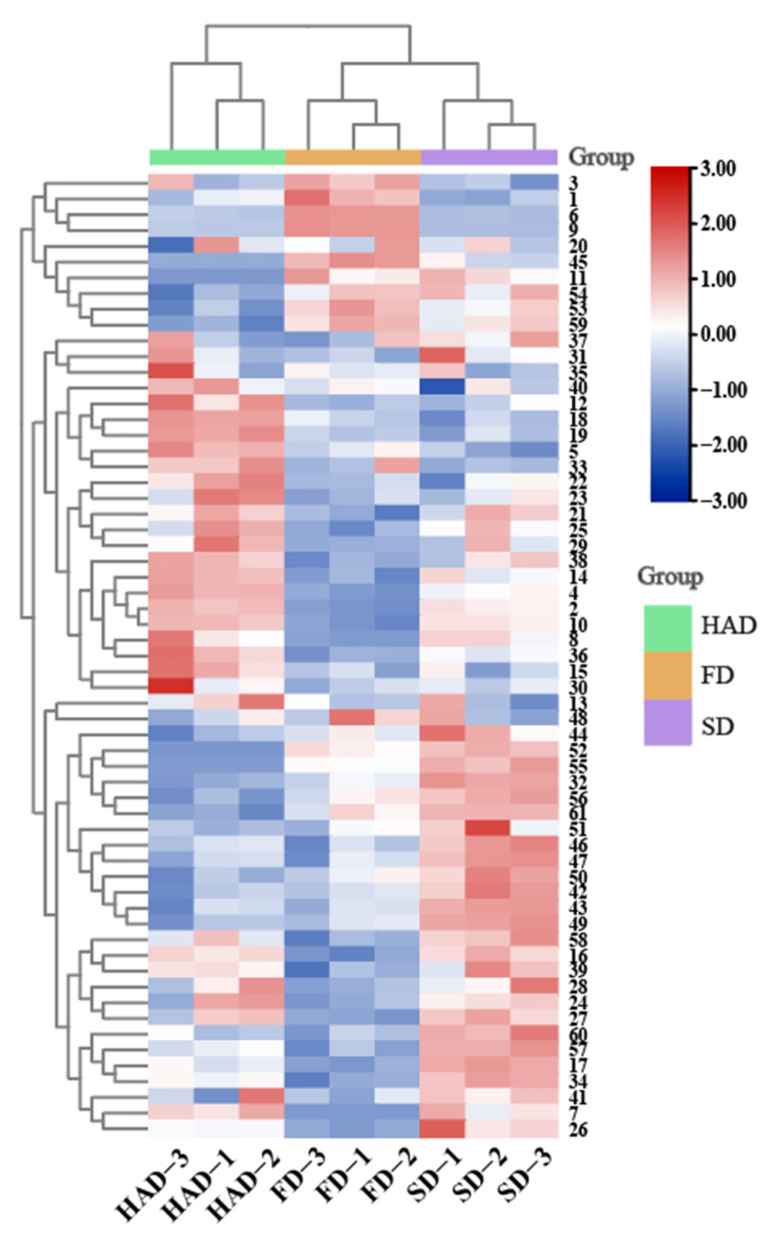
Cluster heat map of volatile components of broad beans treated with three different drying methods.

More accurate insight into broad beans VOCs affected by various drying methods brings a better view of the possibility of applying drying a as preservation method. A total of 61 possible VOCs were identified in broad beans. In this experiment, the volatile organic compounds included 20 hydrocarbons, 10 alcohols, 5 ketones, 10 esters, 6 terpenes, 4 aldehydes, and 6 other compounds (Table S1). As shown in the Figure 2, there was a difference in the percentage of volatile component species in different dried samples, with the specific statistical information for single VOCs in Figure 3.

In summary, the flavor substances of broad beans were made by various types of substances together. Then, alcohols, esters, and aldehydes were the main volatile flavor substances of broad beans. The alkanes were the most abundant in the volatile component, and it was possible that the hydrocarbon compounds were changed during the drying process when the moisture was gradually reduced, resulting in the generation of multiple alkanes [37]. The alcohols were high in 1-hexanol and 1-octen-3-ol, which are aliphatic unsaturated alcohols with mushroom, lavender, rose, and hay aromas. Among the esters, γ-n-amylbutyrolactone had a coconut milk flavor and butterscotch flavor. 2-octen-1-al and 2-butyl-2-octenal also provided flavor contributions. In addition, the VOCs content of broad beans in different drying methods showed different phenomena. FD and HAD could promote the production of alcohols and aldehydes to a great extent, and SD could preserve the esters better. The analysis of its highest content in SD was due to the fact that esters were mainly the products of the esterification reactions of acids and alcohols; milder temperatures made the esters reach the appropriate generation conditions [38]. Because of the milder conditions of HAD and SD, there was no conversion of alcohol, but the Merad reaction still promoted the production of small amounts of aldehydes. However, FD slowed down the chemical reaction because of the lower temperature, so that the VOCs that were not stable enough were preserved.

### 3.5. Total Phenol Content (TPC) and Antioxidant Capacity (AC) Analysis

**Figure 4:**
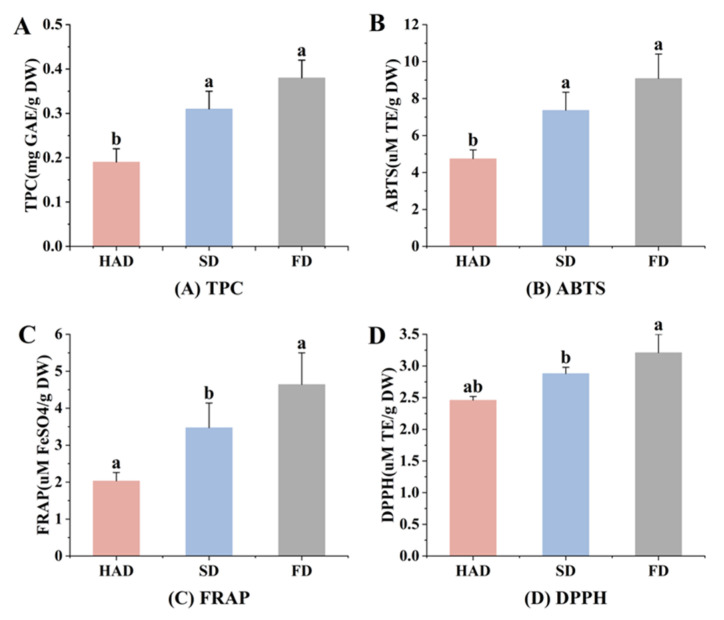
Comparison of total phenol contents and antioxidant activities of broad beans after different drying methods. (**A**) Total phenol content. (**B**) ABTS cation radical scavenging activity. (**C**) Ferric reducing antioxidant power assay. (**D**) DPPH radical scavenging activity. Different superscript letters represent significant differences between different treatments (*p* < 0.05).

The TPC of the dried broad beans are presented in Figure 4A. The results showed that the TPC differed significantly among the dried samples. Compared with FD group (0.38 mg GAE/g DW), the HAD and SD groups decreased by 50% (0.19 mg GAE/g DW) and 18% (0.31 mg GAE/g DW), respectively. FD group was observed the highest TPC value in dried broad beans, which was consistent with the findings of Mcsweeney et al. [39]. This decrease in the TPC of tea by heat treatment might be due to the thermal decomposition of antioxidant components [40]. Many researchers have showed that the SD group has a lower efficiency of preserving the TPC than the HAD group [41], which is different from the phenomena mentioned above. It was reported that a higher rate of non-enzymatic browning may lead to the formation of phenolic substances under a milder temperature condition, such as SD, thereby the negative effects of SD on TPC were lower than that of HAD [42].

The antioxidant capacity of different dried broad beans is compared, and the results are shown in Figure 2. It can be observed that the FD group showed the highest scavenging capacity against different free radicals, and had the strongest ferric reducing ability as well (Figure 2B–D). Generally, the antioxidant activities of broad beans by different drying methods followed the trend of FD > SD > HAD, which indicates that HAD can reduce the TPC and antioxidant capacity of broad beans at varying degrees. Among all drying methods, FD was recommended for optimizing TPC and antioxidant activities.

### 3.6. Identification of Bioactive Substances

**Figure 5:**
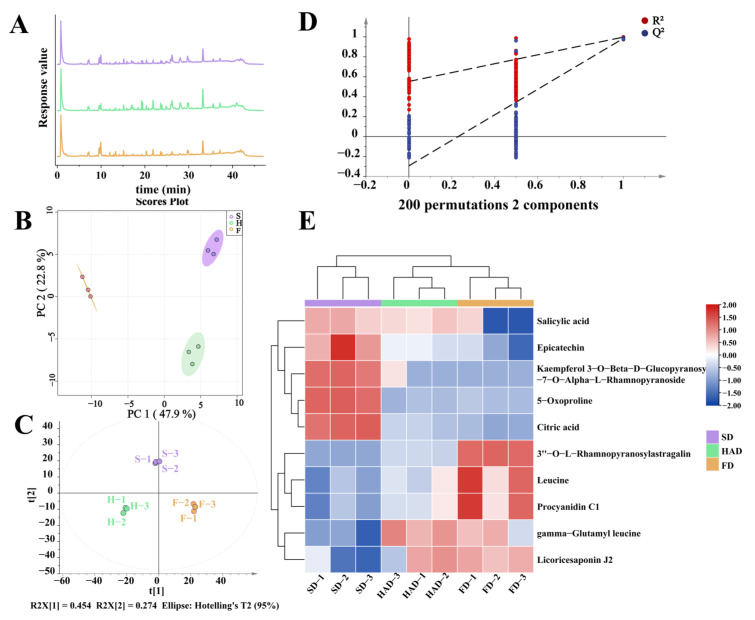
Multivariate statistical analysis showing specificity between three drying methods. (**A**) Total ion chromatograms of the bioactive substance. (**B**) PCA scores plot of the data set. (**C**) PLS-DA scores plot of the data set. (**D**) Random permutation test (200 times) based on the corresponding PLS-DA model. (**E**) Heatmap analysis of differential metabolites contents in broad beans by three drying methods. (H: hot air drying; S: sun drying; F: freeze drying).

The above results showed that different drying methods had obvious effects on the TPC and AC of broad beans. However, further study is needed to understand more about the changes in bioactive substances. The results showed that the total ion chromatograms of bioactive substances by UPLC-Q-TOF-MS were similar among the three drying methods (Figure 5A). A comprehensive chemical profile was provided by unsupervised analysis based on analytical tool measurement of UPLC-Q-TOF/MS. In addition, PCA and PLS-DA were applied to discriminate dried broad beans on the basis of the bioactive substances, which were determined by UPLC-Q-TOF-MS. PCA was applied to distinguish the bioactive substances of dried broad beans among various drying methods, and to analyze possible clustering (Figure 5B). The PCA score plot described that the first two principal components, PC1 (47.9%) and PC2 (22.8%), were a cumulative 70.7%. The bioactive substances of dried broad beans by HAD and SD could be clearly distinguished from the one of FD in PC1. Meanwhile, the substances of dried broad beans by HAD could be distinguished from the others in PC2. Therefore, there was a clear separation in bioactive substances among the broad beans treated by three drying methods.

The PLS-DA model was constructed as a predictive and discriminant model to examine significant sample separation dried technologies for bioactive substances (Figure 5C). R2Y and Q2 are important parameters for verifying the accuracy and reliability of PLS-DA models. In this model, R2Y and Q2 are greater than 0.5, indicating that the model has good predictive power [43]. This model revealed R2X, R2Y, and Q2 values at 0.728, 0.994, and 0.976, respectively. The random permutation test (200 times) proceeded on this PLS-DA model to verify results. The random permutation test (Figure 5D) described that Q2 and R2 values (R2 = [0, 0.619], Q2 = [0, −0.241]) were higher than the real model, resulting in great predictability and goodness of fit. Hence, the above results revealed that the PLS-DA model was repeatable. In summary, statistical tools for unsupervised (PCA) or supervised (PLS-DA) analysis showed statistical differences in bioactive substances among the different drying methods.

Simultaneous ion features of VIP > 1.0 and *p*-value (ANOVA) < 0.05 were considered to be important for the separation of different drying methods according to the standard for the determination of different bioactive substances. Therefore, in this study, certain differential substances were screened based on VIP > 1.0 and *p*-value (ANOVA) < 0.05. The compounds responsible for distinguishing the three types of drying were mainly flavonoids, organic acids, and amino acids (Table S2). The logarithmic conversion data for the 10 labeled compounds were presented in heatmap that visually shows the relative distance of the labeled compounds after drying (Figure 5E). All samples were clearly clustered into three groups, consistent with the PCA results.

Flavonoids of the FD group and SD group were more prominent compared with HAD group, possibly because the heat treatment of HAD oxidized and decomposed the flavonoids. This result was consistent with the findings from previous studies of TPC and AC, and also confirmed the antioxidant effect of certain flavonoids. The flavonoids composed of epicatechin, kaempferol 3-O-beta-D-Glucopyranosyl-7-O-Alpha-L-Rhamnopyranoside, 3″-O-L-Rhamnopyranosylastragalin, and procyanidin C1 have been implicated in these cardiovascular benefits. In addition, the contents of other some amino acids and phenolic acids, such as 5-Oxoproline, Leucine, gamma-Glutamyl leucine, Salicylic acid, and Citric acid in different drying methods were reflected by the heatmap. SD had a good retention effect on those substances because it was not strong enough for mechanical action. The accumulation of some acids after HAD could be attributed to a rising temperature increasing the metabolic rate [44]. Different drying methods had different effects on bioactive components due to their different water loss rates and heat transfer modes [45]. Therefore, the higher content of bioactive substances in FD and SD may be due to less damage caused by lower temperature, which was consistent with the above results of total phenol content and antioxidant activity.

### 3.7. Phenolic Compounds Analysis by UPLC-QQQ-MS

**Figure 6:**
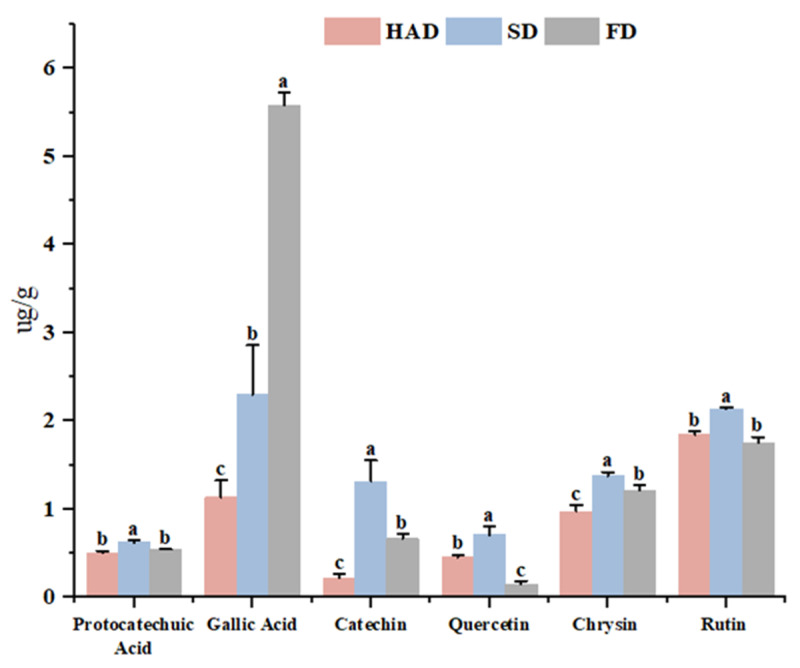
Individual phenolic compounds on dried broad beans. (Different superscript letters of the same substance represent significant differences between different treatments (*p* < 0.05)).

To further elaborate on the variation of phenolic compounds in broad beans, Figure 6 depicts the effect of drying methods on the distribution of individual phenolic compounds in broad beans. Gallic acid was identified as the most abundant phenolic compound with ug/g, reflecting the trend in the overall total TPC and AC, respectively. The gallic acid content increased significantly (*p* < 0.05) after FD treatment compared to HAD and SD, showing a similar phenomenon in rose hips. However, the content of protocatechuic acid, catechin, quercetin, chrysin, and rutin was relatively higher after SD treatment. Regarding the significant decrease in phenolic compounds content after SD treatment (*p* < 0.05), it was speculated that these compounds may be involved in the heat treatment induced by the Maillard reaction.

## 4. Conclusions

In this study, we compared the impact of various drying methods on the nutrient composition, flavor compounds, and primary antioxidant functional components of broad beans. Among the three drying techniques assessed, FD was found to be effective in preserving starch, amino acids, and TPC, exhibiting the strongest AC and highest levels of phenolic compounds, followed by SD. Both FD and HAD significantly promoted alcohol and aldehyde production, while SD better preserved esters. Therefore, this study suggests that FD may be a promising method for food preservation due to its ability to maintain the physical properties, antioxidant potential, and bioactive substances of broad beans. However, considering cost issues, SD could serve as an alternative method. Further research is needed to investigate the impacts of different drying treatments on subsequent processing characteristics.

## Figures and Tables

**Table 1:** Nutrition composition in broad beans using different drying methods.

Drying Method	Moisture (%)	Ash (%)	Starch (%)	Resistant Starch (%)	Protein (%)	Soluble Sugar (%)
HAD	10.49 ± 0.11 ^a^	3.17 ± 0.27 ^a^	40.38 ± 0.88 ^a^	17.75 ± 0.73 ^a^	35.07 ± 0.12 ^a^	17.17 ± 0.17 ^b^
SD	9.80 ± 0.12 ^b^	3.45 ± 0.27 ^a^	39.79 ± 1.95 ^a^	17.30 ± 0.50 ^a^	34.18 ± 0.18 ^b^	18.30 ± 0.02 ^a^
FD	5.68 ± 0.18 ^c^	3.34 ± 0.28 ^a^	40.43 ± 0.38 ^a^	16.87 ± 1.46 ^a^	33.92 ± 0.04 ^b^	15.30 ± 0.28 ^c^

Different superscript letters in the same column represent significant differences between different treatments (*p* < 0.05).

**Table 2:** Amino acids in broad beans using different drying methods.

Amino Acids	Content (mg/g)		
	HAD	SD	FD
Leu *	27.38 ± 0.12 ^b^	28.12 ± 0.22 ^a^	27.95 ± 0.19 ^a^
Lys *	24.22 ± 0.14 ^b^	24.85 ± 0.24 ^a^	24.37 ± 0.14 ^b^
Val *	15.46 ± 0.10 ^b^	15.77 ± 0.11 ^a^	15.89 ± 0.15 ^a^
Phe *	13.55 ± 0.13 ^b^	13.93 ± 0.08 ^a^	13.66 ± 0.08 ^b^
Ile *	13.16 ± 0.06 ^b^	13.43 ± 0.06 ^a^	13.56 ± 0.16 ^a^
Thr *	10.92 ± 0.06 ^b^	11.20 ± 0.10 ^a^	11.08 ± 0.00 ^a^
Met *	1.39 ± 0.07 ^a^	1.29 ± 0.14 ^a^	1.39 ± 0.07 ^a^
Glu	53.54 ± 0.32 ^b^	55.26 ± 0.73 ^a^	53.99 ± 0.12 ^b^
Asp	36.03 ± 0.13 ^b^	34.92 ± 0.25 ^c^	37.53 ± 0.11 ^a^
Arg	33.45 ± 0.25 ^b^	32.29 ± 0.16 ^c^	34.09 ± 0.16 ^a^
Ser	14.43 ± 0.05 ^a^	14.75 ± 0.43 ^a^	14.57 ± 0.10 ^a^
Ala	14.14 ± 0.08 ^a^	14.73 ± 0.11 ^b^	15.59 ± 0.07 ^a^
Gly	13.37 ± 0.11 ^b^	13.84 ± 0.15 ^a^	13.17 ± 0.09 ^b^
Tyr	10.15 ± 0.15 ^b^	10.69 ± 0.15 ^a^	10.33 ± 0.00 ^b^
Pro	7.75 ± 0.53 ^a^	7.75 ± 0.22 ^a^	7.90 ± 0.27 ^a^
His	6.41 ± 0.07 ^a^	6.78 ± 0.19 ^a^	6.57 ± 0.15 ^a^
Cys	0.73 ± 0.00 ^b^	0.89 ± 0.06 ^a^	0.73 ± 0.00 ^b^
Total	296.07	300.48	302.35

* Different superscript letters in the same row represent significant differences between different treatments (*p* < 0.05).

## Data Availability

Data is contained within the article.

## Supplementary Materials

The following supporting information can be downloaded at: https://www.mdpi.com/article/10.3390/foods12112160/s1, Figure S1: The actual photo drying equipment. (A) The convective oven (model GZX-9420MBE, Shanghai Boxun Experimental Co. LTD, China). (B) The freeze-dryer (model RS-232, Labconco Corporation, America).; Table S1: Volatile components of broad beans by different drying methods.; Table S2: Compounds with *p*-value < 0.05 and VIP > 1.0 between broad beans by three drying methods based on the PLS-DA.
