# Supplementary material for: Effects of Drying Treatments on Nutritional Compositions, Volatile Flavor Compounds, and Bioactive Substances of Broad Beans
Source: Foods. 2023 May 26;12(11):2160. doi: 10.3390/foods12112160 (PMC10252730; doi:10.3390/foods12112160)
Supplement: Supplementary file 1 [file foods-12-02160-s001.zip › foods-2389213-supplementary.pdf]

Table S1. Volatile components of broad beans by different drying methods.

NO	Name	CAS	RT	RIx	RI	Elemental composition	Content（%）		
							HAD	SD	FD
1	Lansoprazole	103577-45-3	6.874	821	-	$C_{16}H_{14}F_3N_3O_2S$	0.79±0.13	0.59±0.09	1.40±0.19
2	1-Hexanol	111-27-3	9.027	872	868	$C_6H_{14}O$	20.27±1.14	14.71±0.92	4.87±0.35
3	cis-Hept-2-enal	57266-86-1	13.459	956	958	$C_7H_{12}O$	2.44±0.73	1.85±0.25	3.57±0.24
4	(E)-2-Hepten-1-ol	33467-76-4	14.591	975	978	$C_7H_{14}O$	1.97±0.13	1.16±0.09	0.38±0.07
5	1-Octen-3-ol	3391-86-4	15.198	986	985	$C_8H_{16}O$	6.47±0.42	3.69±0.41	4.67±0.41
6	2-n-Pentylfuran	3777-69-3	15.564	992	993	$C_9H_{14}O$	1.07±0.12	0.76±0.05	11.66±0.44
7	2-Ethyl-6-methylpyrazine	13925-03-6	15.937	998	1003	$C_7H_{10}N_2$	0.32±0.05	0.28±0.09	nd
8	Methylcyclooctane	1502-38-1	16.531	1007	999	C_9H_{18}	0.43±0.07	0.39±0.03	0.23±0.01
9	(+)-m-Mentha-1(6),8-diene	1461-27-4	17.808	1025	1027	$C_{10}H_{16}$	1.90±0.05	1.43±0.03	21.60±1.00
10	trans-3-Octen-2-one	18402-82-9	18.767	1039	1033	$C_8H_{14}O$	4.72±0.21	3.81±0.12	1.59±0.16
11	Benzyl alcohol	100-51-6	19.043	1043	1036	C_7H_8O	nd	0.46±0.10	0.48±0.15
12	(E)-2-Octen-1-al	2548-87-0	20.023	1057	1060	$C_8H_{14}O$	1.49±0.26	0.80±0.15	0.67±0.05
13	2-Methyldecane	6975-98-0	20.299	1061	1064	$C_{11}H_{24}$	0.30±0.04	0.24±0.06	0.23±0.02
14	(2Z)-2-Octen-1-ol	26001-58-1	21.079	1072	1067	$C_8H_{16}O$	3.35±0.34	2.62±0.28	1.65±0.17
15	1-Octanol	111-87-5	21.293	1075	1071	$C_8H_{18}O$	3.28±0.34	2.36±0.33	2.21±0.18
16	trans-Linalool oxide (furanoid)	34995-77-2	22.080	1086	1086	$C_{10}H_{18}O_2$	0.69±0.03	0.74±0.07	0.28±0.04
17	3,5-Octadien-2-one	38284-27-4	22.681	1095	1091	$C_8H_{12}O$	0.93±0.08	1.40±0.04	0.60±0.04
18	O-Decylhydroxylamine	29812-79-1	23.033	1100	1100	$C_{10}H_{23}NO$	0.70±0.02	0.31±0.07	0.39±0.04
19	5-Undecyne	2294-72-6	23.268	1102	1107	$C_{11}H_{20}$	1.02±0.05	0.38±0.11	0.43±0.03
20	Nonanal	124-19-6	23.496	1105	1104	$C_9H_{18}O$	1.61±0.25	1.63±0.11	1.71±0.15
21	10-Undecenyl phenylacetate #	-	24.621	1116	-	$C_{19}H_{28}O_2$	2.38±0.23	2.26±0.37	1.40±0.17
22	Pentylcyclohexane	4292-92-6	25.642	1126	1135	$C_{11}H_{22}$	0.63±0.06	0.45±0.09	0.42±0.03
23	5-Methylundecane	1632-70-8	28.134	1150	1156	$C_{12}H_{26}$	2.32±0.43	1.78±0.22	1.53±0.15
24	2,3-Dimethyldecane	17312-44-6	29.032	1159	1157	$C_{12}H_{26}$	2.69±0.65	2.67±0.12	1.78±0.14
25	3-Methylundecane	1002-43-3	29.701	1165	1170	$C_{12}H_{26}$	2.08±0.31	1.94±0.17	1.37±0.09
26	(4E)-4-Dodecene	7206-15-7	30.633	1174	1181	$C_{12}H_{24}$	3.53±0.02	5.18±1.36	2.15±0.10
27	Dodecane	112-40-3	33.236	1200	1200	$C_{12}H_{26}$	3.58±0.46	3.95±0.19	2.67±0.07
28	2,6-Dimethylundecane	17301-23-4	34.927	1219	1210	$C_{13}H_{28}$	2.07±0.54	2.25±0.53	1.33±0.10
29	(4Z)-4-Tridecene	41446-54-2	39.041	1267	1279	$C_{13}H_{26}$	0.34±0.03	0.29±0.04	0.24±0.00
30	2,6,11-Trimethyldodecane	31295-56-4	39.690	1274	1275	$C_{15}H_{32}$	1.01±0.43	0.60±0.06	0.50±0.08
31	2-Butyl-1-octanol	3913-02-8	40.276	1281	1277	$C_{12}H_{26}O$	0.57±0.11	0.63±0.11	0.47±0.03
32	2-Hexyl-1-octanol	19780-79-1	40.684	1286	-	$C_{14}H_{30}O$	1.52±0.21	2.62±0.45	1.90±0.11
33	(Cetyloxymethyl)oxirane	15965-99-8	41.367	1294	-	$C_{19}H_{38}O2$	0.42±0.03	0.25±0.01	0.32±0.09
34	Tridecane	629-50-5	41.885	1300	1300	$C_{13}H_{28}$	1.98±0.05	2.38±0.09	1.47±0.10
35	1-Dodecene oxide	2855-19-8	42.264	1308	1307	$C_{12}H_{24}O$	0.27±0.06	0.24±0.04	0.26±0.01
36	Pentanoic acid, 10-undecenyl ester	-	42.824	1322	-	$C_{16}H_{30}O_2$	1.58±0.22	1.12±0.05	0.77±0.06
37	2,3,5,8-Tetramethyldecane	192823-15-7	43.134	1329	1318	$C_{14}H_{30}$	0.31±0.06	0.36±0.03	0.30±0.05
38	γ-n-Amylbutyrolactone	104-61-0	44.839	1369	1363	$C_9H_{16}O_2$	3.14±0.25	2.37±0.58	1.29±0.17
39	3-Methyltridecane	6418-41-3	45.019	1373	1371	$C_{14}H_{30}$	1.40±0.03	1.50±0.22	0.97±0.11
40	2-Butyl-2-octenal	13019-16-4	45.191	1378	1378	$C_{12}H_{22}O$	0.71±0.12	0.42±0.19	0.56±0.05
41	(4Z)-4-Tetradecene	41446-65-5	45.606	1387	1378	$C_{14}H_{28}$	0.27±0.06	0.30±0.01	0.24±0.02
42	(3Z)-3-Tetradecene	41446-67-7	45.854	1393	1384	$C_{14}H_{28}$	0.52±0.06	0.86±0.07	0.59±0.03

43	Tetradecane	629-59-4	46.13	1400	1400	$C_{14}H_{30}$	4.48±0.69	7.43±0.20	4.74±0.46
44	Nonylcyclopentane	2882-98-6	47.656	1448	1452	$C_{14}H_{28}$	0.42±0.06	0.75±0.11	0.57±0.05
45	trans-Geranylacetone	3796-70-1	47.925	1456	1453	$C_{13}H_{22}O$	nd	0.26±0.12	0.86±0.10
46	4-(2,6,6-Trimethyl-1-cyclohexenyl)-3-buten-2-one	-	48.912	1488	1491	$C_{13}H_{20}O$	0.75±0.07	1.27±0.12	0.63±0.14
47	2-Hexyl-1-decanol	2425-77-6	49.126	1494	1504	$C_{16}H_{34}O$	0.87±0.14	1.71±0.13	0.87±0.22
48	Ergotamine	113-15-5	49.747	1499	-	$C_{33}H_{35}N_5O_5$	0.29±0.05	0.30±0.09	0.38±0.09
49	2-Hexyl-1-octanol	2882-96-4	51.218	1571	1570	$C_{16}H_{34}$	0.77±0.13	1.76±0.06	0.96±0.12
50	3-Methylpentadecane	544-76-3	51.970	1599	1600	$C_{16}H_{34}$	1.53±0.21	3.02±0.34	2.08±0.24
51	Hexadecane	6785-23-5	53.316	1655	1660	$C_{16}H_{32}$	0.26±0.02	0.45±0.11	0.31±0.05
52	Undecylcyclopentane	6418-44-6	55.960	1771	1770	$C_{18}H_{38}$	nd	0.33±0.02	0.24±0.03
53	2-Hexyl-1-octanol	124-06-1	56.498	1795	1794	$C_{16}H_{32}O_2$	0.14±0.05	0.31±0.05	0.41±0.04
54	3-Methylheptadecane	-	56.581	1799	1804	$C_{16}H_{30}O_3$	0.17±0.03	0.29±0.04	0.28±0.03
55	Tetradecanoic acid, ethyl ester	110-27-0	57.161	1827	1827	$C_{17}H_{34}O_2$	nd	0.40±0.04	0.23±0.01
56	Carbonic acid, tridecyl vinyl ester	502-69-2	57.589	1847	1844	$C_{18}H_{36}O$	0.27±0.05	0.68±0.04	0.49±0.07
57	Isopropyl myristate	54833-48-6	58.659	1899	1889	$C_{21}H_{44}$	0.68±0.07	1.10±0.06	0.40±0.09
58	Hexahydrofarnesyl acetone	112-39-0	59.225	1928	1926	$C_{17}H_{34}O_2$	0.68±0.11	0.86±0.08	0.43±0.07
59	2,6,10,15-Tetramethylheptadecane	628-97-7	60.557	1996	1993	$C_{18}H_{36}O_2$	0.66±0.17	1.93±0.37	2.48±0.34
60	.n-Hexadecanoic acid methyl ester	2462-85-3	62.462	2097	2101	$C_{19}H_{34}O_2$	0.52±0.13	1.09±0.12	0.40±0.10
61	Ethyl hexadecanoate	544-35-4	63.663	2164	2162	$C_{20}H_{36}O_2$	0.44±0.10	2.02±0.01	1.34±0.30

* -: nothing; nd: the substance is not detected.

Table S2. Compounds with P-value < 0.05 and VIP > 1.0 between broad beans by three drying methods based on the PLS-DA.

NO	RT (min)	Proposed compound	M/Z	Elemental composition	Ion	M/Z (Fragment ion)	Error (ppm)
1	1.44	5-Oxoproline	128.03	$C_5H_7NO_3$	$[M-H]^-$	85.0318	0.78
2	1.57	Leucine	130.08	$C_6H_{13}NO_2$	$[M-H]^-$	86.4219	3.07
3	5.86	Salicylic acid	137.02	$C_7H_6O_3$	$[M-H]^-$	93.0365	3.65
4	1.22	Citric acid	191.02	$C_6H_8O_7$	$[M-H]^-$	111.0102;129.0204;173.0088	1.57
5	6.06	gamma-Glutamyl leucine	259.13	$C_{11}H_{20}N_2O_5$	$[M-H]^-$	84.0448;198.1126	5.01
6	3.87	Epicatechin	289.07	$C_{15}H_{14}O_6$	$[M-H]^-$	109.0277;125.0271;203.0692;245.0817	1.73
7	13.34	Kaempferol 3-O-Beta-D-Glucopyranosyl-7-O-Alpha-L-Rhamnopyranoside	593.15	$C_{27}H_{30}O_{15}$	$[M-H]^-$	433.0986	1.52
8	14.35	3''-O-L-Rhamnopyranosylastragalin	593.16	$C_{27}H_{30}O_{15}$	$[M-H]^-$	284.0356;285.0399	2.70
9	17.21	Licoricesaponin J2	823.41	$C_{42}H_{64}O_{16}$	$[M-H]^-$	175.0232;193.0382	1.45
10	8.37	Procyanidin C1	865.19	$C_{45}H_{38}O_{18}$	$[M-H]^-$	125.0288;289.0768;407.0763	-1.73

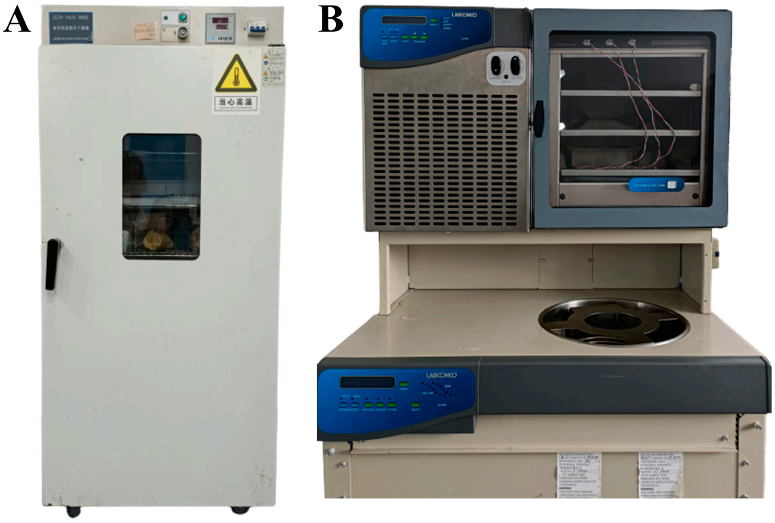

Figure S1. The actual photo drying equipment. (A) The convective oven (model GZX-9420MBE, Shanghai Boxun Experimental Co. LTD, China). (B) The freeze-dryer (model RS-232, Labconco Corporation, America).
